# (*E*)-3-[(3-Eth­oxy-2-hy­droxy­benzyl­idene)amino]­benzoic acid

**DOI:** 10.1107/S1600536812009968

**Published:** 2012-03-10

**Authors:** Hadi Kargar, Zahra Sharafi, Reza Kia, Muhammad Nawaz Tahir

**Affiliations:** a Department of Chemistry, Payame Noor University, PO BOX 19395-3697 Tehran, I. R. of IRAN; b Department of Chemistry, Marvdasht Branch, Islamic Azad University, Marvdasht, Iran; c Department of Chemistry, Science and Research Branch, Islamic Azad University, Tehran, Iran; d Department of Physics, University of Sargodha, Punjab, Pakistan

## Abstract

In the title compound, C_16_H_15_NO_4_, a potential bidentate *N*,*O*-donor Schiff base ligand, the benzene rings are inclined to one another by 4.24 (12)°. The mol­ecule has an *E* conformation about the C=N bond. An intra­molecular O—H⋯N hydrogen bond makes an *S*(6) ring motif. In the crystal, pairs of O—H⋯O hydrogen bonds link the mol­ecules, forming inversion dimers with *R*_2_^2^(8) ring motifs. These dimers are further connected by C—H⋯O inter­actions, forming a sheet in (104). There is also a C—H⋯π inter­action present involving neighbouring mol­ecules.

## Related literature

For background to Schiff bases ligands and their metal complexes, see: Kargar *et al.* (2011 ▶, 2012 ▶); Kia *et al.* (2010 ▶). For standard bond lengths, see: Allen *et al.* (1987 ▶). For hydrogen-bond motifs, see: Bernstein *et al.* (1995 ▶).
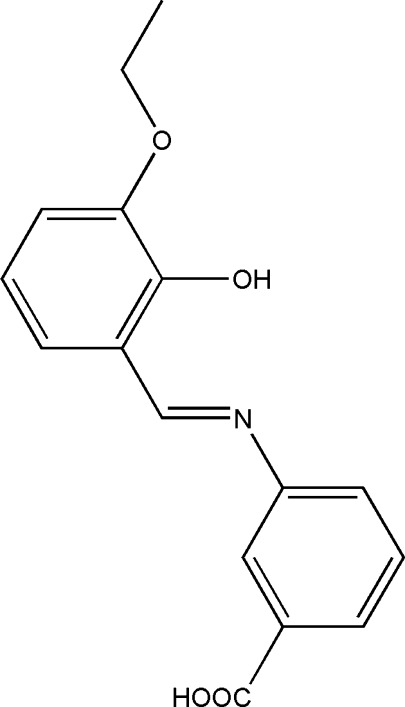


## Experimental

### 

#### Crystal data

C_16_H_15_NO_4_
*M* *_r_* = 285.29
Triclinic, 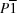

*a* = 5.0306 (3) Å
*b* = 7.1847 (4) Å
*c* = 19.6856 (13) Å
α = 94.956 (4)°
β = 93.310 (4)°
γ = 102.299 (4)°
*V* = 690.45 (7) Å^3^
*Z* = 2
Mo *K*α radiation
μ = 0.10 mm^−1^
*T* = 296 K
0.22 × 0.12 × 0.08 mm

#### Data collection

Bruker SMART APEXII CCD area-detector diffractometer
Absorption correction: multi-scan (*SADABS*; Bruker, 2005 ▶) *T*_min_ = 0.979, *T*_max_ = 0.992
11954 measured reflections
3331 independent reflections
1429 reflections with *I* > 2σ(*I*)
*R*_int_ = 0.058

#### Refinement

*R*[*F* ^2^ > 2σ(*F* ^2^)] = 0.061
*wR*(*F* ^2^) = 0.150
*S* = 0.95
3331 reflections
191 parameters
H-atom parameters constrained
Δρ_max_ = 0.15 e Å^−3^
Δρ_min_ = −0.20 e Å^−3^

### 

Data collection: *APEX2* (Bruker, 2005 ▶); cell refinement: *SAINT* (Bruker, 2005 ▶); data reduction: *SAINT*; program(s) used to solve structure: *SHELXS97* (Sheldrick, 2008 ▶); program(s) used to refine structure: *SHELXL97* (Sheldrick, 2008 ▶); molecular graphics: *SHELXTL* (Sheldrick, 2008 ▶); software used to prepare material for publication: *SHELXTL* and *PLATON* (Spek, 2009 ▶).

## Supplementary Material

Crystal structure: contains datablock(s) global, I. DOI: 10.1107/S1600536812009968/su2387sup1.cif

Structure factors: contains datablock(s) I. DOI: 10.1107/S1600536812009968/su2387Isup2.hkl

Supplementary material file. DOI: 10.1107/S1600536812009968/su2387Isup3.cml

Additional supplementary materials:  crystallographic information; 3D view; checkCIF report

## Figures and Tables

**Table 1:** Hydrogen-bond geometry (Å, °) *Cg*2 is the centroid of the C9–C14 ring.

*D*—H⋯*A*	*D*—H	H⋯*A*	*D*⋯*A*	*D*—H⋯*A*
O3—H3*A*⋯N1	0.99	1.75	2.570 (3)	138
O1—H1*A*⋯O2^i^	0.99	1.63	2.610 (2)	174
C3—H3*B*⋯O1^ii^	0.93	2.58	3.453 (3)	157
C4—H4*A*⋯O2^iii^	0.93	2.53	3.341 (3)	146
C15—H15*A*⋯*Cg*2^iv^	0.97	2.75	3.610 (3)	148

Symmetry codes: (i) 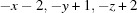; (ii) 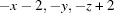; (iii) 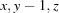; (iv) 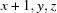.

**Fig. 1.**
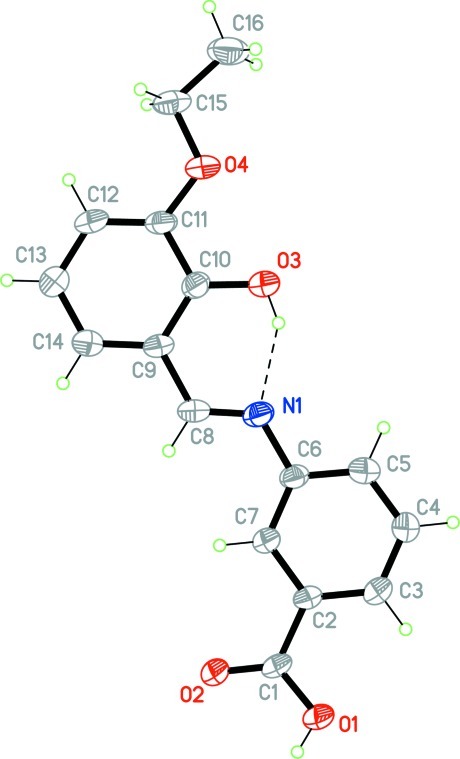
A view of the molecular structure of the title compound, showing 40% probability displacement ellipsoids and the atomic numbering. The dashed line shows the intramolecular O-H···N hydrogen bond - see Table 1 for details.

**Fig. 2.**
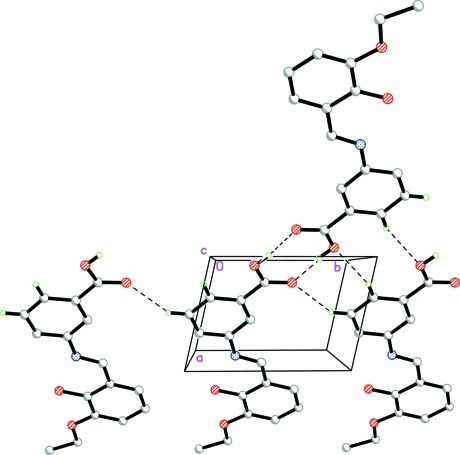
The crystal packing of the title compound, viewed along the c-axis, showing the inversion dimers, with an R22(8) ring motif, which are further connected through C—H···O interactions along the b-axis direction - see Table 1 for details.

## supplementary crystallographic information

### Comment

In continuation of our work on the crystal structure analysis of Schiff base ligands (Kargar *et al.*, 2011, 2012; Kia *et al.*, 2010), we synthesized and determined the crystal structure of the new title potential bidentate N,O-donor Schiff base.

The molecular structure of the title compound is illustrated in Fig. 1. The bond lengths (Allen *et al.*, 1987) and angles are within the normal ranges. The intramolecular O3—H3A···N1 hydrogen bond (Table 1) makes an S(6) ring motif (Bernstein *et al.*, 1995). The dihedral angle between the benzene rings is 4.24 (12)°. The molecule has an E conformation about the C8═N1 bond.

In the crystal, pairs of O—H···O hydrogen bonds (Table 1) link molecules to form inversion dimers with an *R^2^_2_(8)* ring motif. These dimers are connected further by C—H···O interactions along the *b* axis direction, forming a sheet (Fig. 2). There is also a C-H···π interaction present involving neighbouring molecules (Table 1).

### Experimental

The title compound was synthesized by adding 3-ethoxysalicylaldehyde (2 mmol) to a solution of 3-carboxyaniline (2 mmol) in ethanol (30 ml). The mixture was refluxed with stirring for 30 min. The resultant solution was filtered. Pale yellow single crystals of the title compound, suitable for *X*-ray structure determination, were obtained by recrystallization from ethanol, by slow evaporation of the solvents at room temperature over several days.

### Refinement

The O-bound hydrogen atoms were located in a difference Fourier map and constrained to ride on the parent atoms with U_iso_(H) = 1.5 U_eq_(O). The rest of the hydrogen atoms were included in calculated positions and treated as riding atoms: C—H = 0.93, 0.96 and 0.97 Å for CH, CH_3_ and CH_2_ H atoms, respectively, with U_iso_ (H) = k × U_eq_(C), where k = 1.5 for CH_3_ H atoms, and = 1.2 for other H atoms. A rotating group model was applied to the methyl group.

### Crystal data

**Table:**

C_16_H_15_NO_4_	*Z* = 2
*M**_r_* = 285.29	*F*(000) = 300
Triclinic, *P*1	*D*_x_ = 1.372 Mg m^−^^3^
Hall symbol: -P 1	Mo *K*α radiation, λ = 0.71073 Å
*a* = 5.0306 (3) Å	Cell parameters from 2760 reflections
*b* = 7.1847 (4) Å	θ = 2.6–27.7°
*c* = 19.6856 (13) Å	µ = 0.10 mm^−^^1^
α = 94.956 (4)°	*T* = 296 K
β = 93.310 (4)°	Block, pale-yellow
γ = 102.299 (4)°	0.22 × 0.12 × 0.08 mm
*V* = 690.45 (7) Å^3^	

### Data collection

**Table:**

Bruker SMART APEXII CCD area-detector diffractometer	3331 independent reflections
Radiation source: fine-focus sealed tube	1429 reflections with *I* > 2σ(*I*)
Graphite monochromator	*R*_int_ = 0.058
φ and ω scans	θ_max_ = 28.1°, θ_min_ = 2.1°
Absorption correction: multi-scan (*SADABS*; Bruker, 2005)	*h* = −6→6
*T*_min_ = 0.979, *T*_max_ = 0.992	*k* = −9→9
11954 measured reflections	*l* = −25→25

### Refinement

**Table:**

Refinement on *F*^2^	Primary atom site location: structure-invariant direct methods
Least-squares matrix: full	Secondary atom site location: difference Fourier map
*R*[*F*^2^ > 2σ(*F*^2^)] = 0.061	Hydrogen site location: inferred from neighbouring sites
*wR*(*F*^2^) = 0.150	H-atom parameters constrained
*S* = 0.95	*w* = 1/[σ^2^(*F*_o_^2^) + (0.0577*P*)^2^] where *P* = (*F*_o_^2^ + 2*F*_c_^2^)/3
3331 reflections	(Δ/σ)_max_ < 0.001
191 parameters	Δρ_max_ = 0.15 e Å^−^^3^
0 restraints	Δρ_min_ = −0.20 e Å^−^^3^

### Special details

**Table:**

Geometry. All esds (except the esd in the dihedral angle between two l.s. planes) are estimated using the full covariance matrix. The cell esds are taken into account individually in the estimation of esds in distances, angles and torsion angles; correlations between esds in cell parameters are only used when they are defined by crystal symmetry. An approximate (isotropic) treatment of cell esds is used for estimating esds involving l.s. planes.
Refinement. Refinement of F^2^ against ALL reflections. The weighted R-factor wR and goodness of fit S are based on F^2^, conventional R-factors R are based on F, with F set to zero for negative F^2^. The threshold expression of F^2^ > 2sigma(F^2^) is used only for calculating R-factors(gt) etc. and is not relevant to the choice of reflections for refinement. R-factors based on F^2^ are statistically about twice as large as those based on F, and R- factors based on ALL data will be even larger.

### Fractional atomic coordinates and isotropic or equivalent isotropic displacement parameters (Å^2^)

**Table:**

	*x*	*y*	*z*	*U*_iso_*/*U*_eq_	
C1	−0.7756 (4)	0.3599 (4)	0.95116 (13)	0.0401 (6)	
C2	−0.6137 (4)	0.2435 (3)	0.91234 (12)	0.0381 (6)	
C3	−0.6380 (5)	0.0522 (3)	0.92180 (13)	0.0491 (7)	
H3B	−0.7556	−0.0044	0.9526	0.059*	
C4	−0.4873 (5)	−0.0532 (4)	0.88541 (14)	0.0581 (8)	
H4A	−0.5024	−0.1818	0.8916	0.070*	
C5	−0.3131 (5)	0.0305 (4)	0.83961 (13)	0.0514 (7)	
H5A	−0.2132	−0.0429	0.8149	0.062*	
C6	−0.2848 (5)	0.2212 (4)	0.82988 (12)	0.0394 (6)	
C7	−0.4376 (5)	0.3274 (3)	0.86627 (12)	0.0419 (6)	
H7A	−0.4226	0.4559	0.8599	0.050*	
C8	−0.0261 (5)	0.4639 (4)	0.77162 (12)	0.0450 (7)	
H8A	−0.1074	0.5525	0.7949	0.054*	
C9	0.1761 (4)	0.5293 (3)	0.72375 (12)	0.0404 (6)	
C10	0.3028 (5)	0.3975 (3)	0.68892 (13)	0.0418 (6)	
C11	0.4947 (5)	0.4626 (4)	0.64210 (13)	0.0461 (7)	
C12	0.5568 (5)	0.6515 (4)	0.63127 (13)	0.0515 (7)	
H12A	0.6851	0.6933	0.6006	0.062*	
C13	0.4304 (5)	0.7824 (4)	0.66551 (14)	0.0559 (8)	
H13A	0.4725	0.9106	0.6574	0.067*	
C14	0.2425 (5)	0.7208 (4)	0.71145 (13)	0.0516 (7)	
H14A	0.1590	0.8084	0.7346	0.062*	
C15	0.7861 (5)	0.3715 (4)	0.55931 (14)	0.0554 (8)	
H15A	0.9426	0.4700	0.5781	0.067*	
H15B	0.6947	0.4196	0.5221	0.067*	
C16	0.8758 (6)	0.1926 (4)	0.53405 (16)	0.0740 (9)	
H16A	1.0043	0.2217	0.5002	0.111*	
H16B	0.7201	0.0979	0.5142	0.111*	
H16C	0.9603	0.1441	0.5717	0.111*	
N1	−0.0939 (4)	0.2889 (3)	0.78237 (10)	0.0452 (6)	
O1	−0.9217 (3)	0.2754 (2)	0.99589 (9)	0.0551 (5)	
H1A	−1.0308	0.3568	1.0191	0.083*	
O2	−0.7718 (3)	0.5279 (2)	0.93998 (9)	0.0527 (5)	
O3	0.2460 (3)	0.2104 (2)	0.69762 (9)	0.0583 (5)	
H3A	0.0815	0.1769	0.7229	0.087*	
O4	0.6037 (4)	0.3213 (2)	0.61093 (9)	0.0613 (6)	

### Atomic displacement parameters (Å^2^)

**Table:**

	*U*^11^	*U*^22^	*U*^33^	*U*^12^	*U*^13^	*U*^23^
C1	0.0324 (14)	0.0490 (16)	0.0379 (16)	0.0025 (11)	0.0121 (11)	0.0089 (12)
C2	0.0340 (14)	0.0450 (16)	0.0359 (16)	0.0074 (11)	0.0103 (11)	0.0064 (12)
C3	0.0483 (16)	0.0466 (17)	0.0541 (19)	0.0064 (12)	0.0205 (13)	0.0137 (14)
C4	0.0690 (19)	0.0396 (16)	0.071 (2)	0.0141 (14)	0.0290 (16)	0.0115 (14)
C5	0.0498 (16)	0.0517 (18)	0.056 (2)	0.0152 (13)	0.0189 (13)	0.0039 (14)
C6	0.0334 (13)	0.0479 (16)	0.0394 (16)	0.0106 (11)	0.0117 (11)	0.0085 (12)
C7	0.0420 (14)	0.0428 (15)	0.0440 (17)	0.0104 (11)	0.0149 (12)	0.0104 (12)
C8	0.0422 (15)	0.0557 (18)	0.0397 (17)	0.0146 (12)	0.0152 (12)	0.0024 (13)
C9	0.0340 (14)	0.0496 (16)	0.0395 (17)	0.0100 (11)	0.0123 (11)	0.0058 (12)
C10	0.0374 (14)	0.0463 (17)	0.0442 (17)	0.0102 (12)	0.0107 (11)	0.0119 (13)
C11	0.0409 (15)	0.0562 (18)	0.0459 (18)	0.0161 (12)	0.0183 (12)	0.0077 (13)
C12	0.0415 (16)	0.0612 (19)	0.0542 (19)	0.0099 (13)	0.0232 (13)	0.0103 (14)
C13	0.0570 (18)	0.0477 (17)	0.064 (2)	0.0069 (13)	0.0199 (15)	0.0106 (15)
C14	0.0528 (17)	0.0498 (18)	0.054 (2)	0.0133 (13)	0.0196 (14)	0.0005 (14)
C15	0.0464 (16)	0.080 (2)	0.0456 (18)	0.0209 (14)	0.0204 (13)	0.0118 (15)
C16	0.068 (2)	0.084 (2)	0.073 (2)	0.0216 (17)	0.0306 (17)	−0.0062 (17)
N1	0.0406 (12)	0.0528 (15)	0.0460 (15)	0.0125 (10)	0.0169 (10)	0.0111 (11)
O1	0.0545 (11)	0.0557 (12)	0.0635 (13)	0.0175 (8)	0.0359 (9)	0.0186 (9)
O2	0.0548 (11)	0.0448 (11)	0.0638 (13)	0.0126 (8)	0.0298 (9)	0.0149 (9)
O3	0.0578 (12)	0.0537 (12)	0.0724 (15)	0.0200 (9)	0.0338 (10)	0.0179 (10)
O4	0.0587 (12)	0.0651 (12)	0.0676 (14)	0.0196 (9)	0.0376 (10)	0.0117 (10)

### Geometric parameters (Å, º)

**Table:**

C1—O2	1.242 (3)	C10—O3	1.342 (2)
C1—O1	1.289 (3)	C10—C11	1.409 (3)
C1—C2	1.482 (3)	C11—C12	1.365 (3)
C2—C3	1.384 (3)	C11—O4	1.370 (3)
C2—C7	1.389 (3)	C12—C13	1.392 (3)
C3—C4	1.369 (3)	C12—H12A	0.9300
C3—H3B	0.9300	C13—C14	1.377 (3)
C4—C5	1.379 (3)	C13—H13A	0.9300
C4—H4A	0.9300	C14—H14A	0.9300
C5—C6	1.378 (3)	C15—O4	1.426 (3)
C5—H5A	0.9300	C15—C16	1.505 (3)
C6—C7	1.380 (3)	C15—H15A	0.9700
C6—N1	1.420 (3)	C15—H15B	0.9700
C7—H7A	0.9300	C16—H16A	0.9600
C8—N1	1.270 (3)	C16—H16B	0.9600
C8—C9	1.458 (3)	C16—H16C	0.9600
C8—H8A	0.9300	O1—H1A	0.9862
C9—C14	1.391 (3)	O3—H3A	0.9867
C9—C10	1.403 (3)		
			
O2—C1—O1	122.6 (2)	C9—C10—C11	119.0 (2)
O2—C1—C2	121.30 (19)	C12—C11—O4	125.9 (2)
O1—C1—C2	116.1 (2)	C12—C11—C10	120.2 (2)
C3—C2—C7	120.1 (2)	O4—C11—C10	113.9 (2)
C3—C2—C1	120.2 (2)	C11—C12—C13	120.8 (2)
C7—C2—C1	119.7 (2)	C11—C12—H12A	119.6
C4—C3—C2	119.4 (2)	C13—C12—H12A	119.6
C4—C3—H3B	120.3	C14—C13—C12	119.6 (2)
C2—C3—H3B	120.3	C14—C13—H13A	120.2
C3—C4—C5	120.3 (2)	C12—C13—H13A	120.2
C3—C4—H4A	119.9	C13—C14—C9	120.9 (2)
C5—C4—H4A	119.9	C13—C14—H14A	119.6
C6—C5—C4	121.1 (2)	C9—C14—H14A	119.6
C6—C5—H5A	119.4	O4—C15—C16	107.0 (2)
C4—C5—H5A	119.4	O4—C15—H15A	110.3
C5—C6—C7	118.7 (2)	C16—C15—H15A	110.3
C5—C6—N1	114.9 (2)	O4—C15—H15B	110.3
C7—C6—N1	126.4 (2)	C16—C15—H15B	110.3
C6—C7—C2	120.4 (2)	H15A—C15—H15B	108.6
C6—C7—H7A	119.8	C15—C16—H16A	109.5
C2—C7—H7A	119.8	C15—C16—H16B	109.5
N1—C8—C9	121.7 (2)	H16A—C16—H16B	109.5
N1—C8—H8A	119.2	C15—C16—H16C	109.5
C9—C8—H8A	119.2	H16A—C16—H16C	109.5
C14—C9—C10	119.5 (2)	H16B—C16—H16C	109.5
C14—C9—C8	120.8 (2)	C8—N1—C6	123.1 (2)
C10—C9—C8	119.8 (2)	C1—O1—H1A	113.1
O3—C10—C9	122.6 (2)	C10—O3—H3A	110.9
O3—C10—C11	118.3 (2)	C11—O4—C15	117.80 (19)
			
O2—C1—C2—C3	−174.8 (2)	C14—C9—C10—C11	−0.1 (4)
O1—C1—C2—C3	4.2 (3)	C8—C9—C10—C11	179.1 (2)
O2—C1—C2—C7	5.1 (4)	O3—C10—C11—C12	179.4 (2)
O1—C1—C2—C7	−175.9 (2)	C9—C10—C11—C12	0.3 (4)
C7—C2—C3—C4	0.0 (4)	O3—C10—C11—O4	−0.6 (3)
C1—C2—C3—C4	179.9 (2)	C9—C10—C11—O4	−179.7 (2)
C2—C3—C4—C5	−0.1 (4)	O4—C11—C12—C13	179.3 (3)
C3—C4—C5—C6	0.6 (4)	C10—C11—C12—C13	−0.7 (4)
C4—C5—C6—C7	−0.9 (4)	C11—C12—C13—C14	0.7 (4)
C4—C5—C6—N1	178.6 (2)	C12—C13—C14—C9	−0.4 (4)
C5—C6—C7—C2	0.8 (4)	C10—C9—C14—C13	0.1 (4)
N1—C6—C7—C2	−178.7 (2)	C8—C9—C14—C13	−179.0 (2)
C3—C2—C7—C6	−0.3 (4)	C9—C8—N1—C6	178.4 (2)
C1—C2—C7—C6	179.8 (2)	C5—C6—N1—C8	−173.9 (2)
N1—C8—C9—C14	178.4 (2)	C7—C6—N1—C8	5.6 (4)
N1—C8—C9—C10	−0.7 (4)	C12—C11—O4—C15	−4.1 (4)
C14—C9—C10—O3	−179.1 (2)	C10—C11—O4—C15	175.9 (2)
C8—C9—C10—O3	0.0 (4)	C16—C15—O4—C11	179.8 (2)

### Hydrogen-bond geometry (Å, º)

Cg2 is the centroid of the C9–C14 ring.

**Table:**

*D*—H···*A*	*D*—H	H···*A*	*D*···*A*	*D*—H···*A*
O3—H3*A*···N1	0.99	1.75	2.570 (3)	138
O1—H1*A*···O2^i^	0.99	1.63	2.610 (2)	174
C3—H3*B*···O1^ii^	0.93	2.58	3.453 (3)	157
C4—H4*A*···O2^iii^	0.93	2.53	3.341 (3)	146
C15—H15*A*···*Cg*2^iv^	0.97	2.75	3.610 (3)	148

Symmetry codes: (i) −*x*−2, −*y*+1, −*z*+2; (ii) −*x*−2, −*y*, −*z*+2; (iii) *x*, *y*−1, *z*; (iv) *x*+1, *y*, *z*.
